# Impact of Threshold Setting for Event Log Repair on Conformance Checking

**DOI:** 10.1155/tswj/4028269

**Published:** 2025-03-31

**Authors:** Kazuki Masumoto, Hiroki Horita

**Affiliations:** College of Engineering, Ibaraki University, Hitachi, Japan

**Keywords:** business analysis, business information systems, conformance checking, data quality, process mining

## Abstract

Conformance checking is a method to compare the actually executed business process recorded as an event log with the business process described as a business process model and to identify differences. For human or technical reasons, event logs that contain noise and are of low quality may be recorded. Therefore, methods have been proposed to repair low-quality event logs, but they require the setting of a threshold, and it is difficult to set a suitable threshold. In this paper, we investigate the effect of low-quality event log repair methods on conformance checking. Through experiments, it was shown that the appropriate threshold depends on the type of event log and the amount of noise.

## 1. Introduction

Modeling business processes is becoming increasingly important in the development of information systems [1]. The validity of business processes, that is, whether they satisfy the properties required by the business, has a significant impact on the usefulness of information systems [2]. To analyze and model business processes, interviews and observational surveys are conducted with business personnel, and documents such as business manuals are checked. These methods are problematic in that they are subjective, provide only fragmentary information, and require a great deal of time. Therefore, process mining [3] is attracting attention as an objective, comprehensive, and rapid analysis method to solve these problems. Process mining is an attempt to understand the business processes actually executed by analyzing the event logs of the information systems used in an organization's operations. For example, process discovery methods [4] can be used to automatically build business process models from event logs to facilitate understanding of how work flows. In addition, conformance checking [5] is a major analysis method in process mining. This method compares event logs, which are records of actually executed business processes, with normative business process models that describe the flow of work to identify similarities and differences and can be used to find unfavorable dissociations that suggest fraud or inefficiency [3].

The reliability of the results of the process mining is strongly influenced by the quality of the event logs [6]. Reference [6] defines five levels of maturity for the quality of event log data, from excellent quality (⋆⋆⋆⋆⋆) to low quality (⋆). In principle, process mining techniques are considered to be applicable to ⋆⋆⋆⋆⋆, ⋆⋆⋆⋆, and ⋆⋆⋆. This means that many process mining methods assume that the recorded event data is reliable [7]. Using event logs with data quality issues without careful consideration can lead to counterintuitive or misleading process mining results and can lead to detrimental business decisions [8]. However, due to human errors such as omissions or technical problems such as differences in timestamp formats due to the use of multiple information systems, only low-quality event logs containing noise such as missing or abnormal values may be obtained [9, 10]. Here, the less noise is included in the event log, the higher the quality of the event log is considered. To cope with this problem, various methods have been proposed to repair anomalies by preprocessing the event logs before process mining [11]. The impact of event log quality on the task of process discovery, in which business process models are generated from event logs without prerequisite knowledge, has been investigated so far [12]. However, the impact of event log quality on conformance checking, one of the main tasks of process mining, has not been investigated.

In this paper, we investigate how low-quality event logs containing noise affect conformance checking. Specifically, we performed conformance checking after repairing event logs with various threshold values used in the Sani et al. repair method [13] and compared the results. The experiments were performed on event logs that contained various proportions of noise (the noise we are dealing with here is noise from the addition of random events, the deletion of random events, and the replacement of random events).

The remainder of this paper is organized as follows: Section 2 describes related works.  Section 3 provides background knowledge on conformance checking and event log repair. In Section 4, we present our experiments and results on how event log repair threshold settings affect conformance checking. Finally, Section 5 concludes the paper and discusses future work.

## 2. Related Works

Data quality has long been a topic of interest in the fields of statistics and data mining [14]. Data come in a variety of formats, and preprocessing methods are required for each format. Therefore, this section describes research on the quality of event logs.

Most of the papers on event log data quality are concerned with methods to repair noise. Various methods have been proposed for repairing missing values and repairing timestamps [15, 16]. The method used in this paper by Sani et al. [13] was used because it was developed with the intention of being used as a preprocessor for a wide range of analyses using process mining.

In addition to noise repair, other studies including the extraction of missing tendency [17], defect detection [18], and quality evaluation [19–23] have been studied for the detection and evaluation of problems in event logs. Determining thresholds for repair methods after evaluating the quality of event logs using these methods is a future challenge.

Pegoraro et al. proposed a conformance-checking method for uncertain data [24]. In their paper, conformance checking is performed with the uncertain part explicitly indicated. On the other hand, we assume that the uncertain part is not known in advance.

## 3. Background

### 3.1. Conformance Checking

Conformance checking is a method to compare event logs, which are records of actually executed business processes, and normative business process models, which describe the flow of business operations, to identify similarities and differences. This paper uses an alignment-based conformance-checking method [15]. With this method, the business process model and the event log can be replayed in parallel, and the correspondence between events in the event log and events in the business process model can be checked one by one. In order to verify that traces (an ordered list of events and accompanying information executed from the start to the end of a business process) conform to the business process model, it is necessary to align them. Therefore, a series of actions are performed to replay the traces in the business process model. This behavior can be divided into three types: synchronous move, model-only move, and log-only move. The synchronous move means that the same event occurs in both the business process model and the event log when they are replayed one at a time in parallel. This is a state in which events in the business process model and event log are synchronized. If different events occur when replaying the business process model and the event log, the business process model and the event log must be moved asynchronously. A model-only move represents an event of a business process model that does not exist in the event log at the position being replayed. A log-only move represents an event that has no counterpart in the event log of the business process model at the location being replayed. The fitness of the trace and the business process model can be obtained from the number of moves, etc. The fitness is expressed as a real number between 0 and 1, and the closer it is to 1, the higher the degree of fit between the trace and the business process model. The details of this algorithm are described in [5].

**Figure 1:**
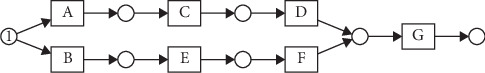
A Petri net–based business process model.

We illustrate conformance checking with a simple event log and an example business process model. The process model is shown in Figure 1. In this model, there is an XOR branch at 1 in the figure, and only one of the paths is executed. The contents of the event logs are shown in Table 1. Traces are distinguished by trace IDs. < B, E, F, G> indicate that the events were executed in order from left to right. Each event may have attributes such as a timestamp and the person who executed it. When the business process model in Figure 1 and trace t1 are replayed in parallel, B occurs first in both, followed by E, F, and G, in turn, in both the process model and trace t1. Since all of these moves occur simultaneously, fitness is maximized to 1. Similarly, in trace t2, all moves occur simultaneously. In trace t3, F is missing. Therefore, there is an event for a business process model that does not exist in the event log, and only the model is moved. In trace t4, H is between C and D. When the next move after C is replayed, there is an event H that does not have a corresponding event in the business process model. In this case, a log-only move occurs. When model-only move and log-only move occur, fitness is lowered because the business process model and the event log do not match.

### 3.2. Event Log Repair

In this paper, we use the method proposed by Sani et al. [11] for repairing event logs. There have been several studies on event log repair methods such as [25, 26]. Among them, Sani et al.'s method [13] was developed with the intention of being used as a preprocessing method for a wide range of process mining analysis. However, the impact on conformance checking, a key process mining technique, has not been confirmed, and this is confirmed in this paper.

The method of Sani et al. [13] uses event occurrence probabilities to detect and repair noise. The probability of occurrence of a subsequence consisting of events in a trace is calculated and considered as noise if it is lower than a set threshold. The event log is then repaired by searching for a subsequence of similar length to the noisy subsequence and replacing the noisy location with the subsequence that has the highest probability among all candidate subsequences. It is not clear how the threshold value should be set to improve the quality of the event log, given the various possible amounts of noise in the event log. This is the motivation for our research.

## 4. Experiments

In this section, we describe experiments to confirm the effect of threshold setting on conformance checking in a low-quality event log repair algorithm, as well as the results and discussion of the experiments. In this paper, experiments were conducted using ProM^1^, an open-source process mining environment.

### 4.1. Experimental Flow

The experimental flow is as follows.

1. Adding noise to the event log: A plug-in called “add, swap, and remove events” is used on ProM to add noise to the event log by deleting, adding, and swapping events. The amount of noise added was varied from 5%, 10%, 20%, and 30% to create event logs containing noise, respectively.
2. Repairing event logs containing noise: The event logs containing noise are repaired using the method of Sani et al. [13]. The repair requires a threshold value to be set, which can be varied, and the results are compared later in the process.
3. Performing conformance checking: Conformance checking is performed using Adriansyah et al.'s method [5]. This method can be used with the plugin “replay a log on Petri net for performance/conformance analysis.” The settings in (1) and (2) are compared in terms of their effects on synchronous move, model-only move, log-only move, and average fitness (the average of the fitness obtained for each trace). Comparisons are made because these are standard measures for interpreting the results of conformance checking.

**Figure 2:**
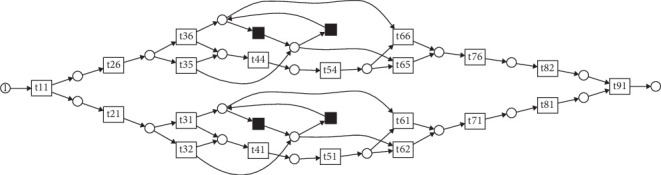
Business process model used in Experiment (a).

**Figure 3:**
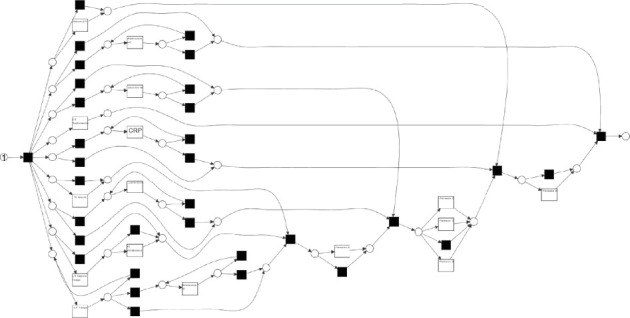
Business process model used in Experiment (b).

The experiments used (a) an artificial event log from the Process Discovery Contest 2020^2^ and (b) a real-life event log of sepsis cases in a hospital^3^. The event log in (a) contains 16029 events in 1000 traces. The business process model is also available on the website.  Figure 2 shows the business process model of (a). The event log in (b) contains 15,000 events in about 1000 traces. The business process model used was created on ProM using the inductive miner [27].  Figure 3 shows the business process model in (b).

### 4.2. Results

#### 4.2.1. Experiments on Event Log (a)

**Figure 4:**
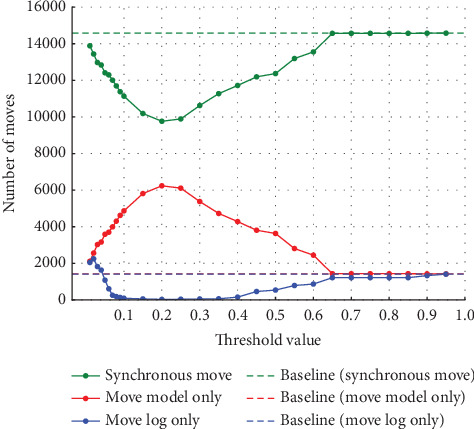
Results at 5% noise in event Log (a).

**Figure 5:**
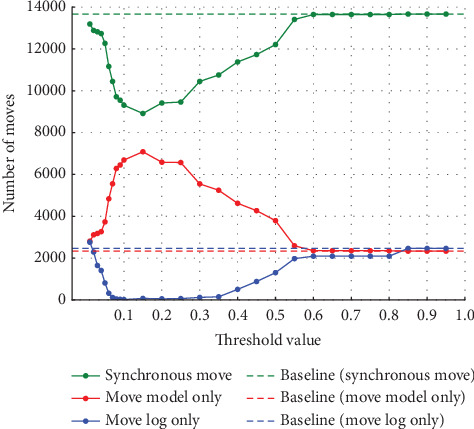
Results at 10% noise in event Log (a).

**Figure 6:**
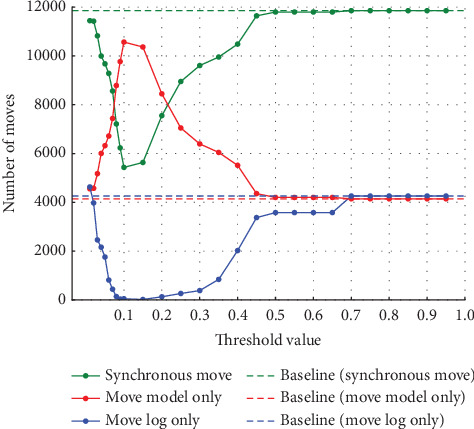
Results at 20% noise in event Log (a).

**Figure 7:**
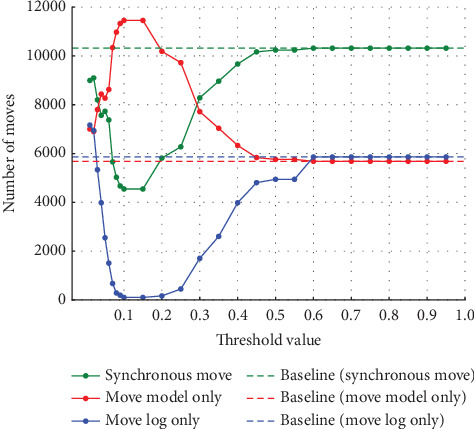
Results at 30% noise in event Log (a).

In Experiment (a), the noise was varied from 5%, 10%, 20%, and 30%. The thresholds set for event log repair varied in 0.01 increments from 0.01 to 0.10, where a large percentage of change was observed, and in 0.05 increments from 0.10 to 0.95. The results for each of the noise settings from 5% to 30% are shown in the four figures from Figures 4, 5, 6, and 7. The vertical axis represents the number of each item (synchronous move, model-only move, and log-only move), and the horizontal axis represents the threshold value. The baseline represented by the dotted line is the result of conformance checking of the event logs with noise added and without repair.

In all figures, the number of synchronous move decreases to a threshold value of 0.15 and then increases until it equals the baseline (synchronous move). For model-only move, the opposite change from synchronous moves is observed. For log-only move, the number of move is close to zero when the threshold value of 0.10 is reached, regardless of the noise ratio. As the noise ratio is varied, the number of synchronous move decreases overall, the number of model-only move increases overall, and the number of log-only move increases overall except in the range approaching zero. When both the number of model-only and log-only moves reach a certain threshold, the number of move becomes equal to the baseline, indicating that no preprocessing has taken place.

**Figure 8:**
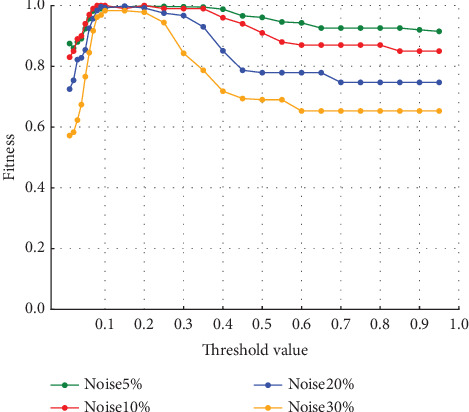
Event Log (a) trace average fitness per percentage of noise.

The average fitness of the traces in the event logs with varying noise ratios is shown in Figure 8. The vertical axis represents the average fitness of the traces, and the horizontal axis represents the threshold value. It can be seen that the average fitness of the traces approaches 1 in the range where the number of log-only move decreases in any ratio. The average fitness of the traces generally decreases as the ratio of noise increases.

#### 4.2.2. Experiments on Event Log (b)

**Figure 9:**
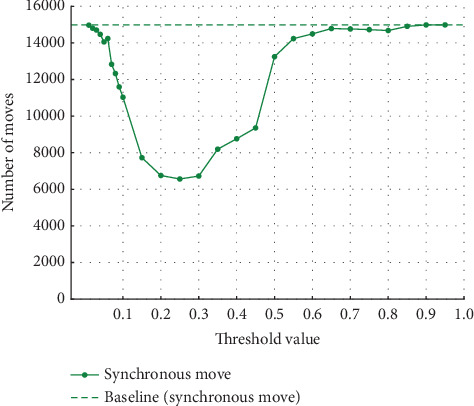
Number of synchronous move at 5% noise in event Log (b).

**Figure 10:**
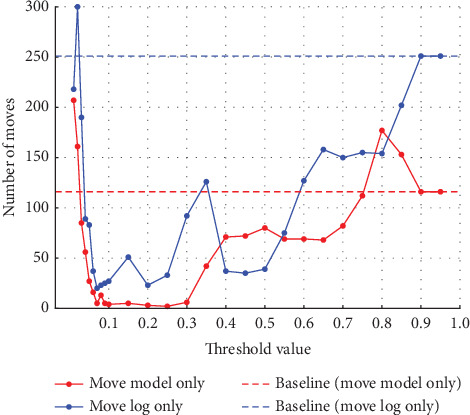
Number of model-only move and log-only move at 5% noise in event Log (b).

**Figure 11:**
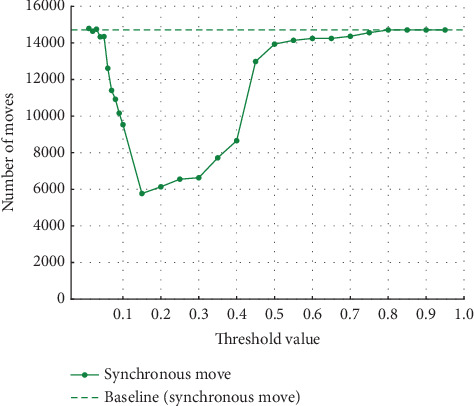
Number of synchronous move at 10% noise in event Log (b).

**Figure 12:**
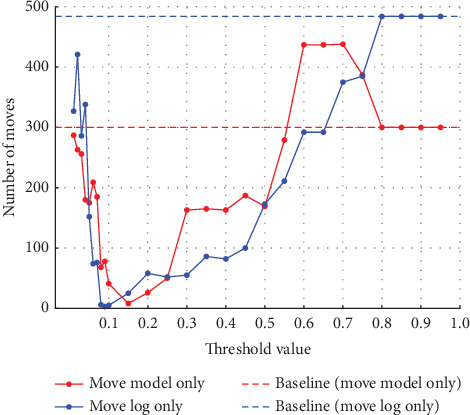
Number of model-only move and log-only move at 10% noise in event Log (b).

**Figure 13:**
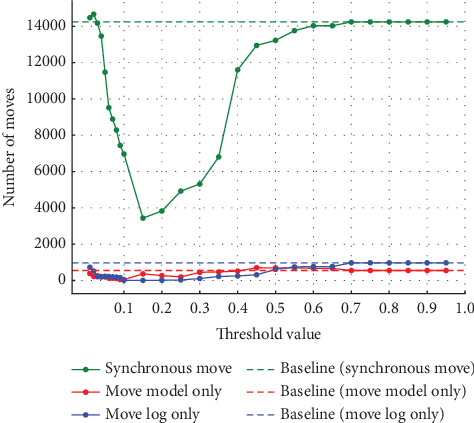
Results at 20% noise in event Log (b).

**Figure 14:**
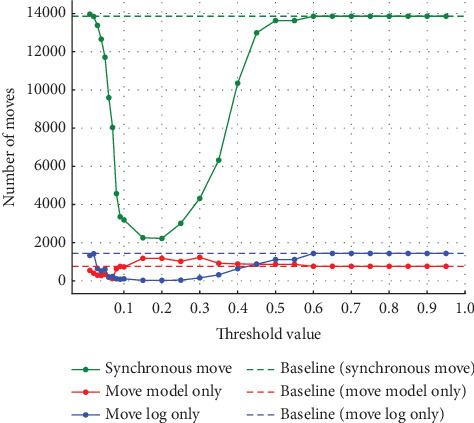
Results at 30% noise in event Log (b).

Similarly, in Experiment (b), the noise was varied by 5%, 10%, 20%, and 30%. As in Experiment (a), the threshold used to detect noise was also varied in 0.01 increments from 0.01 to 0.10 and in 0.05 increments from 0.10 to 0.95.  Figure 9 shows the synchronous move at 5% noise, and Figure 10 shows the model-only and log-only move at 5% noise.  Figure 11 shows the synchronous move at 10% noise, and Figure 12 shows the model-only and log-only move.  Figure 13 shows the results with 20% noise, and Figure 14 shows the results with 30% noise. The vertical axis represents the number of each item (synchronous move, model-only move, and log-only move), and the horizontal axis represents the threshold value. The baseline represented by the dotted line is the result of conformance checking of the event logs with noise added and without repair.

In Experiment (a), the number of model-only move increased in the threshold range from 0 to around 0.15 and then decreased as the threshold approached 1. In Experiment (b), on the other hand, the number of move decreased once in the threshold range from 0 to 0.3, approached 0, and then increased. The number of synchronous and log-only move reached a minimum around the threshold value of 0.15 for all noise ratios. The range over which the number of move decreases and increases is similar to Experiment (a), with the number of log-only move decreasing from threshold 0 to around 0.15 and then increasing. As in Experiment (a), once a certain threshold is reached, it is clear that no preprocessing has taken place.

**Figure 15:**
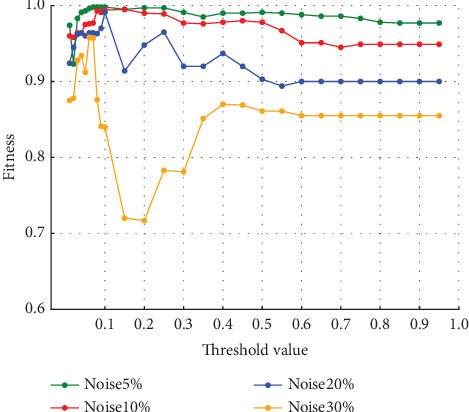
Event Log (b) trace average fitness per percentage of noise.

The trace average fitness for the four-event logs with varying percentages of noise in Experiment (b) is shown in Figure 15. The average fitness of the traces was higher in the range of around the threshold of 0.05–0.25 when the percentage of noise was 5% and 10%. When the noise percentage was 20%, the highest values were obtained at the threshold of 0.1, and when the noise percentage was 30%, the highest values were obtained at the thresholds of 0.06 and 0.07. Unlike in Experiment (a), the average fitness of the traces decreases and increases after reaching its highest value.

### 4.3. Discussion

#### 4.3.1. Discussion of Experiment (a)

Experiment (a) shows that the number of log-only move increases in the range of threshold settings below 0.08 and above 0.3. In this range, the average fitness of the traces decreases. In this range, the number of log-only move increases because values that should be considered noise are not considered noise, indicating that the threshold set to identify noise is inappropriate. If the threshold is too low, the noise may not be considered noise. Since the event with the highest probability of occurrence is replaced as the appropriate event for repair, if the threshold is too high, even the event with the highest probability of occurrence may be recognized as noise, and the event is not replaced; the noise remains. Therefore, in the range of threshold values below 0.08, the accuracy of repair decreases as the value approaches 0, and the number of log-only move increases. Similarly, in the range of threshold values above 0.3, the number of log-only move is considered to increase as it approaches 1. When a certain threshold value is reached, it matches the baseline, which indicates the number in the event logs that have not been repaired, indicating that the repair has not been successful. The range in which the average fitness of the traces is higher is influenced by the noise ratio, but the average fitness of the traces is close to 1 in the range of threshold values from 0.08 to 0.2. In this range, we can see that the repair is successful. The quality of the repaired event logs varies depending on the set threshold value. The experimental results show that the number of log-only move is a major cause of the decrease in the average fitness of the traces and that it also affects the results of the conformance checking.

#### 4.3.2. Discussion of Experiment (b)

In Experiment (a), the model-only move did not affect the average fitness of the traces. However, in Experiment (b), the average fitness of the traces was affected by both model-only and log-only move. Therefore, both model-only and log-only move were lower in the range where the average fitness of the traces was higher. As the percentage of noise increases, the average fitness of the trace increases or decreases significantly. In this event log, the average fitness increases in the range of the threshold value from 0.05 to 0.10 when the noise percentage is between 5% and 10% and reaches its maximum value at the threshold value of 0.10 when the noise percentage is 20%. When the noise percentage is 30%, the values are higher at thresholds of 0.06 and 0.07. In the same event log, the range where the average fitness of the trace becomes high varies depending on the noise percentage. In Experiment (a), the average fitness of the traces reached its maximum value at approximately the threshold value of 0.10, even when the percentage of noise was varied. It can be seen that the range where the average fitness of the trace becomes high varies depending on the event log. Therefore, it is not easy to find the optimal threshold value depending on the type of event log and the noise ratio.

## 5. Conclusion

In this paper, we investigate for the first time how threshold settings for repairing low-quality event logs affect the results of conformance checking. Specifically, we investigated how synchronous move, log-only move, model-only move, and average fitness change by varying the threshold value and the percentage of noise in the event log.

Since the optimal threshold value may vary depending on the type of noise and its tendency [17], further investigation and devising a method to automatically set the threshold value are future works. In these experiments, noise was artificially mixed into the event log, but it is not clear how to accurately determine the percentage of noise in the event log in general. It is possible to utilize a method such as [21] to measure the quality of the event log and then set a threshold for the repair method and perform the repair. In order to generalize the results of this paper, the number of types of event logs used in the experiments should be increased. We would like to consider future work such as selecting a larger number of domains and larger and more complex targets.

## Figures and Tables

**Table 1:** Example of event log.

**Trace ID**	**Contents of trace**
t1	< B, E, F, G>
t2	< A, C, D, G>
t3	< B, E, G>
t4	< A, C, H, D, G>

## Data Availability

The data used in this study are available at the following two links: https://icpmconference.org/2020/process-discovery-contest/data-set/ and https://data.4tu.nl/articles/dataset/Sepsis_Cases_-_Event_Log/12707639/1.

## Endnotes

^1^ https://www.promtools.org/doku.php

^2^ https://icpmconference.org/2020/process-discovery-contest/

^3^ https://data.4tu.nl/articles/dataset/Sepsis_Cases_-_Event_Log/12707639/1
